# Impact of Macroporosity on Catalytic Upgrading of Fast Pyrolysis Bio‐Oil by Esterification over Silica Sulfonic Acids

**DOI:** 10.1002/cssc.201700959

**Published:** 2017-08-16

**Authors:** Jinesh C. Manayil, Amin Osatiashtiani, Alvaro Mendoza, Christopher M.A. Parlett, Mark A. Isaacs, Lee J. Durndell, Chrysoula Michailof, Eleni Heracleous, Angelos Lappas, Adam F. Lee, Karen Wilson

**Affiliations:** ^1^ European Bioenergy Research Institute Aston University Birmingham B4 7ET UK; ^2^ Department of Chemical and Energy Technology Universidad Rey Juan Carlos C/Tulipán s/n, E- 28933 Móstoles Madrid Spain; ^3^ Chemical Process & Energy Resources Institute Centre for Research and Technology-Hellas (CPERI/CERTH) 6th km Harilaou-Thermi Road 57001 Thessaloniki Greece

**Keywords:** acidity, bio-oil, esterification, mesoporous silica, sulfonic acid

## Abstract

Fast pyrolysis bio‐oils possess unfavorable physicochemical properties and poor stability, in large part, owing to the presence of carboxylic acids, which hinders their use as biofuels. Catalytic esterification offers an atom‐ and energy‐efficient route to upgrade pyrolysis bio‐oils. Propyl sulfonic acid (PrSO_3_H) silicas are active for carboxylic acid esterification but suffer mass‐transport limitations for bulky substrates. The incorporation of macropores (200 nm) enhances the activity of mesoporous SBA‐15 architectures (post‐functionalized by hydrothermal saline‐promoted grafting) for the esterification of linear carboxylic acids, with the magnitude of the turnover frequency (TOF) enhancement increasing with carboxylic acid chain length from 5 % (C_3_) to 110 % (C_12_). Macroporous–mesoporous PrSO_3_H/SBA‐15 also provides a two‐fold TOF enhancement over its mesoporous analogue for the esterification of a real, thermal fast‐pyrolysis bio‐oil derived from woodchips. The total acid number was reduced by 57 %, as determined by GC×GC–time‐of‐flight mass spectrometry (GC×GC–ToFMS), which indicated ester and ether formation accompanying the loss of acid, phenolic, aldehyde, and ketone components.

## Introduction

Biofuels have an important role to play in mitigating anthropogenic climate change arising from the combustion of fossil fuels.^1^ In the context of energy, despite significant growth in fossil fuel reserves, great uncertainties remain in the economic and environmental impact of exploitation, and crucially, approximately 65–80 % of such carbon resources cannot be burned without breaching the United Nations framework convention on climate change (UNFCC) target to keep the global temperature rise this century well below 2 °C. Biofuels will prove critical in helping many countries meet their renewable energy commitments, which for the UK are 15 % by 2020, alongside greenhouse gas (GHG) emission reductions of 34 % by 2020 and 80 % by 2050 (compared with 1990 levels). They also represent drop‐in fuels able to utilize existing pipeline and filling station distribution networks.^2^ Thermochemical processing of waste biomass such as lignocellulosic materials sourced from agriculture or municipal waste offers a promising route to biofuels through pyrolysis.^3^

Pyrolysis is a widespread approach for bio‐oil^4^ synthesis, in which biomass is thermally decomposed in an oxygen‐free or oxygen‐limited environment.^5^ The resulting crude bio‐oil is a complex mixture of acids, alcohols, furans, aldehydes, esters, ketones, sugars, and multifunctional compounds such as hydroxyacetic acid, hydroxyl‐acetaldehyde and hydroxyacetone (derived from cellulose and hemicellulose), together with 3‐hydroxy‐3‐methoxy benzaldehyde, phenols, guaiacols, and syringols derived from the lignin component.1b, 6 Pyrolysis bio‐oils thus require “upgrading” through deoxygenation and neutralization to enhance their energy density, stability, and physical properties.6a, 7 A range of catalytic upgrading methods are known,^8^ at least at the laboratory scale, including esterification,^9^ ketonization,^10^ hydrodeoxygenation,^11^ and condensation.^12^

Carboxylic acids comprise 5–10 wt % of pyrolysis bio‐oils,^9^, ^13^ and are largely responsible for their poor chemical stability. Hence, esterification (particularly employing bio‐derived alcohols such as methanol, ethanol, or phenols^9^, ^14^) offers an energy‐efficient and atom‐economical route to upgrading.8b, 15 Homogeneous mineral acid catalysts are historically employed for esterification, however their process disadvantages and poor (environmental) E‐factors are well‐documented; hence, strong drivers remain for the development of heterogeneous solid acid counterparts.^11^ Although base catalysts are widely used for the transesterification of vegetable oils (triacylglycerides) to yield biodiesel, they are unsuitable for catalytic esterification owing to neutralization/saponification.1d

Diverse solid acids have been explored for esterification, including zeolites,^16^ heteropolyacids,^17^ sulfated metal oxides,^18^ carbon‐based acid catalysts,^19^ and functionalized mesoporous silicas.^20^ Research on the latter indicates that mesoporous SBA‐15,^21^ KIT‐6,^22^ and PMO^23^ sulfonic acids, and a macroporous–mesoporous SBA‐15 (MM‐SBA‐15)20g analogue, are among the most promising owing to their tunable pore architecture strong Brønsted acidity and hydrophobicity.2a, 14, 20g, 23, 24 3‐Propylsulfonic acid (PrSO_3_H)/SBA‐15 has been reported as an efficient catalyst for acetic acid esterification with methanol2a, 25 and other alcohols in simulated bio‐oils,^26^ and the most widely used sulfonic acid in solid acid catalyzed esterification.^27^ Such catalysts exhibit improved water tolerance during esterification when the sulfonated silica surface is co‐functionalized with alkyl chains.2a, 5, 25b We recently reported a post‐modification hydrothermal saline‐promoted grafting (HSPG) route to introduce higher sulfonic acid loadings into mesoporous silicas than those achievable by conventional grafting methods,24a and confer stability towards leaching during the esterification of model acids.24b, 28 Hydrophobicity and catalytic reactivity, can also be enhanced through incorporating organic groups into the silica framework.24b Mesopore interconnectivity also plays a role in controlling esterification activity, with interconnectivity between the hexagonal cylindrical mesopores of PrSO_3_H/KIT‐6 offering superior mass transport and active site accessibility to non‐interconnected PrSO_3_H/SBA‐15.20g Mesopore expansion (from ≈5 to 14 nm),^14^ and macropore incorporation^23^ offer alternative approaches to enhance the esterification activity of PrSO_3_H/SBA‐15 for long chain fatty acid esterification.

With respect to bio‐oil upgrading through catalytic esterification, most studies have employed only model compounds owing to the complex nature of real pyrolysis bio‐oils7a and the associated analytical challenge. We previously reported the application of PrSO_3_H/SBA‐15 for acetic acid esterification of model bio‐oils.^26^, ^28^ Here, we report the synthesis and application of HSPG‐derived mesoporous PrSO_3_H/SBA‐15, and a macroporous counterpart, for the esterification of simple carboxylic acids (C_3_, C_6_, and C_12_), and the upgrading of thermal fast pyrolysis bio‐oil derived from woodchips.

## Results and Discussion

### Catalyst characterization

The successful synthesis of an ordered mesoporous skeleton for SBA‐15 and a macroporous–mesoporous (MM) skeleton for MM‐SBA‐15 (with a mean macropore diameter of ≈200 nm, close to that of the polystyrene colloidal hard template, Figure S1 in the Supporting Information) supports was confirmed by TEM. An ordered, 2D hexagonal mesopore channel network was observed for the former, and a well‐defined interconnecting macropore‐mesopore network for the latter (Figure S2). Formation of the desired *p6mm* pore architecture for both SBA‐15 and MM‐SBA‐15 was confirmed by low angle X‐ray diffraction (Figure S3), which revealed reflections characteristic of hexagonally ordered mesostructures. Both supports retained hexagonal close packed pore architectures following functionalization by propylsulfonic acid in a H_2_O/NaCl mixture (the HSPG method). However, a shift in the diffraction peaks to higher angle was observed post‐functionalization owing to mesopore contraction.^23^ Mesopore generation (and retention after sulfonation) was further evidenced by N_2_ porosimetry, which showed type IV isotherms with H1 hysteresis loops for all materials (Figure S4). The textural properties of PrSO_3_H/SBA‐15 and PrSO_3_H/MM‐SBA‐15 are summarized in Table 1. The BET surface areas decreased after sulfonic acid grafting over both silicas owing to micropore blockage, which was apparent as a dramatic drop in the micropore area and pore volume. These changes were accompanied by a decrease in pore diameter and an increase in wall thickness, suggesting the uniform grafting of sulfonic acid groups throughout both pore networks without distortion of their unit cells. Previous studies have shown the macropores in such hierarchical frameworks are open and interconnected by bottleneck pore openings.^23^, ^29^

**Table 1:** Physicochemical properties of mesoporous SBA‐15 and macroporous–mesoporous SBA‐15 and their sulfonic acid analogues.

Sample	Surface area	*d*_p_	*V*_total_	*V*_micropore_	Wall thickness	Unit cell parameter	S loading	Acid loading
	[m^2^ g^−1^]^[a]^	[nm]^[b]^	[cc g^−1^]	[cc g^−1^]^[c]^	[nm]	[nm]	[wt %]^[d]^	[mmol g^−1^]^[e]^
SBA15	879	5.5	1.17	0.08	5.5	11.0	–	–
PrSO_3_H/SBA15	379	3.8	0.49	0.01	7.3	11.1	5.8	1.5
MM‐SBA‐15	357	4.5	0.55	0.02	5.9	9.0	–	–
PrSO_3_H/MM‐SBA‐15	186	3.4	0.24	0.00	7.2	9.2	5.5	1.6

[a] BET, [b] BJH, [c] t‐plot, [d] CHNS, [e] propylamine adsorption/TGA‐MS.

Diffuse reflectance infrared fourier transform spectra (DRIFTS) of the parent silicas showed bands at 700–1400 cm^−1^ and 3000–3800 cm^−1^, which were indicative of framework Si‐*O*‐Si and surface silanols, respectively (Figure S5).^15^ Additional bands appeared at approximately 2960‐2830 cm^−1^after sulfonation of both materials, which were attributed to CH_2_ vibrations of the propyl backbone, and a new CH_2_−Si band centered at 1360 cm^−1^. CHNS elemental analysis of the sulfonated silicas revealed that both contained approximately 6 wt % sulfur (Table 1), which represented a five‐fold increase over conventional toluene grafting,^14^, ^23^ in good agreement with our preliminary results using the HSPG method.24a S 2p XP spectra of both sulfonic‐acid‐functionalized materials in Figure S6 reveal two distinct S chemical environments; a low binding energy centered at 164.5 eV associated with unoxidized thiol, and a higher energy doublet arising from sulfonic acid groups centered at 168.9 eV.^30^ Quantitative XPS analysis (Table S1) showed that approximately 85 % of S was incorporated as sulfonic acid groups. Thermogravimetric analysis (Figure S7 b) highlighted two major weight losses; one below 100 °C, which was attributed to physisorbed water; and the second between 250–650 °C owing to propylsulfonic acid decomposition.^31^ The bulk S content estimated from this second loss feature was approximately 5 wt % in accordance with elemental analysis. Acid properties of both sulfonated silica were subsequently probed through pyridine and propylamine adsorption. DRIFT spectra of pyridine‐titrated materials (Figure S8) evidenced only Brønsted acid sites.^26^ Temperature‐programmed analysis of reactively formed propene from chemisorbed propylamine confirmed that PrSO_3_H/SBA‐15 and PrSO_3_H/MM‐SBA‐15 possessed similar acid strengths and loadings (Figure S9 and Figure S10). Therefore, the incorporation of macropores into the SBA‐15 architecture had minimal impact on silica functionalization; the propylsulfonic acid functions grafted over silica surfaces in PrSO_3_H/SBA‐15 and PrSO_3_H/MM‐SBA‐15 catalysts were chemically identical. Therefore, any differences in TOFs between the two catalysts must arise solely from diffusion phenomena. However, despite their similar acid site loadings, the surface coverage of acid sites was higher over the macroporous material (which possessed a lower surface area). Note that the higher S loadings accessible through the HSPG method offer acid loadings of approximately 1.5 mmol g^−1^, approximately twice those obtained through sulfonic acid grafting in toluene (0.6–0.8 mmol g^−1^).2a Molecular dynamics simulations and adsorption calorimetry revealed that cooperative effects between silanol and sulfonic acid functions can weaken their acidity in PrSO_3_H/MCM‐41 owing to hydrogen bonding and associate sulfonate reorientation.^32^ However, such effects only operated for low sulfonic acid loadings, and were absent on crowded surfaces such as those employed in this work; hence, cooperative effects were not expected to influence the catalytic performance.

### Esterification of model carboxylic acids

The catalytic performance of mesoporous and macroporous–mesoporous sulfonic acid silicas was evaluated in the esterification of propanoic (C_3_), hexanoic (C_6_), and lauric acids (C_12_) with methanol to explore the influence of the macropores on the reactivity under previously optimized conditions.2a Because both catalysts possessed similar acid loadings and strength, any differences in activity must arise from their pore architecture. Both sulfonic acid catalysts were active for methylic esterification of the C_3_, C_6_, and C_12_ acids (Figure S11), which were 100 % selective to their corresponding methyl esters. The rate of esterification decreased with increasing alkyl chain length owing to polar and steric effects.^33^

**Figure 1:**
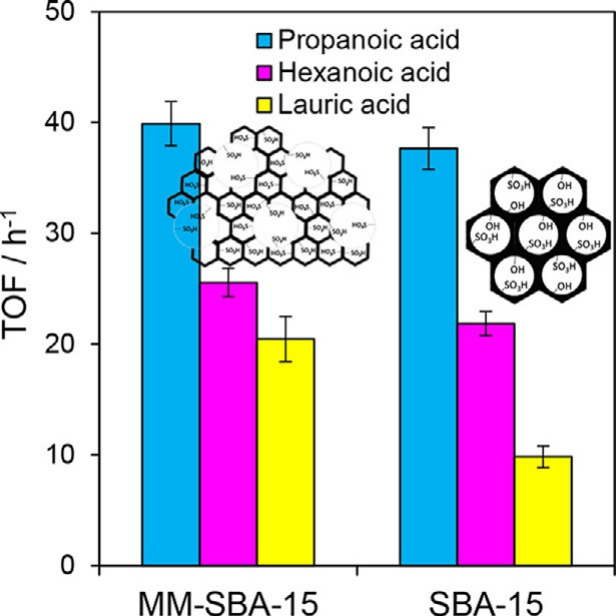
TOF for esterification of various carboxylic acids over PrSO_3_H/SBA‐15 and PrSO_3_H/MM‐SBA‐15 catalysts. (Reaction conditions: 25 mg catalyst, 5 mmol acid, acid/MeOH molar ratio=1:30, 60 °C).

The associated turnover frequencies (TOFs) for carboxylic acid esterification were similar over both catalysts for the C_3_ and C_6_ acids (Figure 1), whereas the TOF for lauric acid over the hierarchical PrSO_3_H/MM‐SBA‐15 was twice that observed for the purely mesoporous PrSO_3_H/SBA‐15 (Figure S12). This rate enhancement for the bulky lauric acid esterification could be explained in terms of improved sulfonic acid accessibility through (i) faster in‐pore diffusion of the reactant/ester product; (ii) shorter mesopore channel lengths owing to truncation by macropores; and (iii) an increased number of mesopore openings, which may boost the sulfonic acid density at mesopore entrances.^23^

### Esterification of thermal pyrolysis bio‐oil

The performance of both sulfonic acid silicas was also assessed for the upgrading of a bio‐oil produced by thermal fast pyrolysis of oak woodchips at a bench‐scale, continuous fluidized bed reactor at 500 °C. Some physicochemical properties of the parent biomass feedstock are presented in Table S2, and of the crude bio‐oil in Table S3. Although the bio‐oil possessed a similar calorific value to the woodchips, the volumetric energy density of the former was significantly higher than that of the original biomass, whose density was only 600–900 kg m^−3^. The bio‐oil contained 23 wt % water, typical of fast pyrolysis bio‐oil,6b, 34 although the total acid number (TAN) of 61.6 mg KOH g^−1^ measured by the Modified D664A acid number titration method^35^ was relatively low.^34^

**Figure 2:**
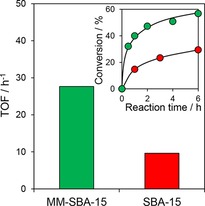
Effect of support architecture on the TOFs of sulfonic acid catalyzed bio‐oil esterification. Inset: acid conversion profiles for bio‐oil esterification using sulfonic acid catalysts. (Reaction conditions: 9.2 g bio‐oil ≈10 mmol acid, 12.1 mL MeOH (acid/MeOH molar ratio=1:30), 100 mg catalyst, 85 °C).

Figure 2 compares TOFs for total acid removal (as determined by KOH titration) through catalytic esterification with methanol, and the corresponding reaction profiles for total acid conversion (Figure 2 inset). The PrSO_3_H/MM‐SBA‐15 catalyst was almost three times more active in terms of TOF, and converted twice the amount of acid than the PrSO_3_H/SBA‐15 after 6 h. Because the pyrolysis oil contains numerous bulky compounds as described in Table 2, Table 3, and Table S4, we attributed the superior performance of the hierarchical catalyst to improved active site accessibility akin to that for lauric acid esterification. The carboxylic acid constituents of fast pyrolysis bio‐oils may drive low level (<5 %) autocatalytic esterification.^36^ This was consistent with a control experiment in the absence of any sulfonic acid catalyst, which revealed <8 % total acid conversion of the pyrolysis bio‐oil. Hence, autocatalysis exerted minimal impact on our results.

**Table 2:** Compositions of crude and upgraded bio‐oils following treatment with PrSO_3_H/MM‐SBA‐15 catalyst.

Group	Crude bio‐oil/ Area [%]	Upgraded bio‐oil/ Area [%]
aromatic hydrocarbons	1.8	1.9
aliphatic hydrocarbons	0.4	2.1
phenolic compounds	25.8	7.8
furanic compounds	0.6	1.4
organic acids	19.7	0.9
esters	1.9	11.8
alcohols	1.1	26.1
ethers	1.0	6.5
aldehydes	5.2	0.4
ketones	10.8	2.9
sugars and anhydro sugars	26.6	13.5
unidentified	5.3	24.7

**Table 3:** Esters present in crude and upgraded thermal fast pyrolysis bio‐oils following treatment with PrSO_3_H/MM‐SBA‐15 catalyst.

Crude bio‐oil	Esterified bio‐oil
acetic acid, methyl ester	acetic acid, methyl ester
formic acid, 2‐propenyl ester	butanedioic acid, dimethyl ester
ethanedioic acid, diethyl ester	hexanoic acid, methyl ester
propanoic acid, ethenyl ester	9‐octadecenoic acid (*Z*)‐, methyl ester
ethyl homovanillate	butanedioic acid, methyl‐, dimethyl ester
	methyl propionate
	octanoic acid, methyl ester
	levulinic acid, methyl ester
	nonanoic acid, methyl ester

**Figure 3:**
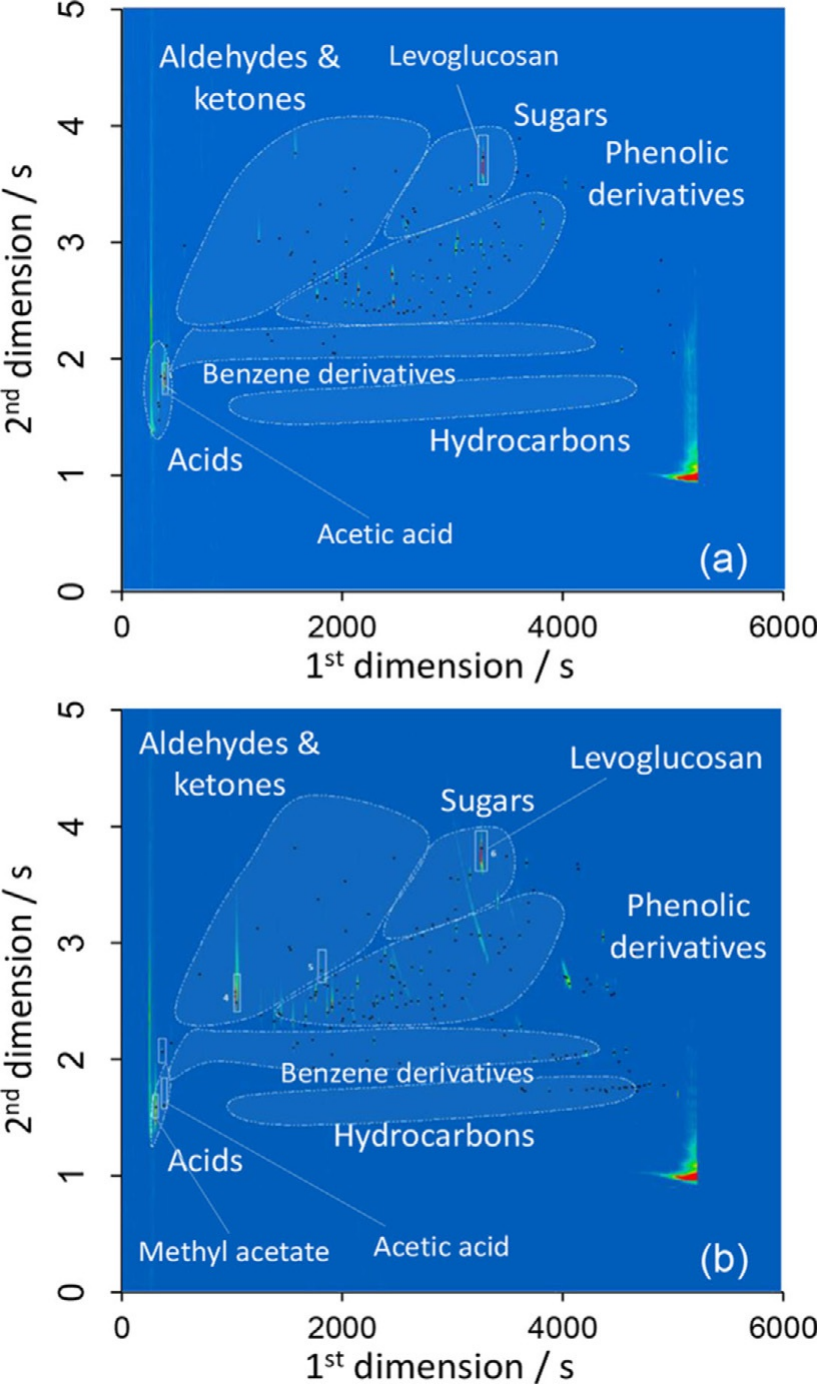
GC×GC–ToFMS chromatogram of a) crude thermal fast pyrolysis bio‐oil and b) bio‐oil after esterification over PrSO_3_H/MM‐SBA‐15.

The chemical composition of the crude and upgraded bio‐oil following catalytic treatment by PrSO_3_H/MM‐SBA‐15 were analyzed in detail by GC×GC–time‐of‐flight mass spectrometry (GC×GC–ToFMS), and the resulting 2D chromatograms are shown in Figure 3. For both the crude and upgraded bio‐oils, the chromatographic space was divided into six discreet molecular groups: acids and esters; aldehydes and ketones (including furanoics and cyclic carbonyls); hydrocarbons (saturated and unsaturated non‐aromatic); aromatic hydrocarbons; phenolic compounds; and sugars. Compounds that could not be identified by the library and/or did not meet the required identification criteria (as detailed in the Supporting Information) were classified as “unidentified”. A more detailed classification of each molecular group and their relative chromatographic area is presented in Table 2. Almost complete loss of organic acids (from 19.7 to 0.9 %) and a significant decrease in phenolics, ketones, aldehydes, and sugars was observed following catalytic upgrading, accompanied by a significant increase in ester and alcohol components, consistent with esterification. Additional details on the removal/formation of specific phenolics, ethers, and carbonyls is presented in Table S4. Acetic acid was the major organic acid in both the crude and upgraded bio‐oils. Esters with relative areas >0.1 in the crude and upgraded bio‐oils are presented in Table 3.

Methyl acetate accounted for 10.8 % of the total chromatographic area of the esterified bio‐oil, as compared to only 1.4 % of the crude bio‐oil, alongside a range of methyl and dimethyl esters from C_3_–C_11_ compounds. Identifiable ethers were mainly C_3_–C_6_ methoxy‐compounds, with 1,1,2,2‐tetramethoxyethane predominant. Considering phenolics, upgrading principally removed methoxy‐phenols, whereas cresol and catechol derivatives were recalcitrant. The increase in alcohols appeared to arise from glycolaldehyde dimethyl acetal (GDA) formation from levoglucosan.^37^ Previous studies have revealed that levoglucosan can be transformed in alcohol media by acid catalysts to methyl levulinate, through intermediate glycolaldehyde (GA) formation^38^ (which may itself form glycolaldehyde dimethyl acetal). GA and GDA were detected in the upgraded bio‐oil, supporting this proposed reaction pathway. Future work will address the recyclability of PrSO_3_H/MM‐SBA‐15 for the esterification of real bio‐oils, wherein we expect strong adsorption of organics that will require the development of low‐temperature regeneration protocols that avoid decomposition of the grafted sulfonate.

In summary, GC×GC–ToFMS analysis confirmed that PrSO_3_H/MM‐SBA‐15 was an effective catalyst for the esterification of a real thermal pyrolysis bio‐oil, significantly reducing the bio‐oil acidity through esterification of organic acids under mild reaction conditions.

## Conclusions

Mesoporous and hierarchical macroporous–mesoporous (MM) propyl sulfonic acid (PrSO_3_H) silicas were synthesized by hydrothermal saline‐promoted grafting of the pre‐formed architectures. The textural properties of the parent silicas were unperturbed by sulfonation, which resulted in similar sulfonic acid loadings and strengths for both pore networks. The turnover frequencies for catalytic esterification of model C_3_–C_12_ carboxylic acids with methanol decreased with alkyl chain length over both materials, however the introduction of 200 nm macropores into the SBA‐15 framework doubled the activity per acid site for the bulkiest lauric acid, which was attributed to enhanced mass transport and active site access, and a higher −PrSO_3_H surface density. Macropore incorporation also enhanced the esterification activity for the upgrading of a real bio‐oil derived from thermal fast pyrolysis of oak woodchips; the TOF for total organic acid removal increased three‐fold relative to the mesoporous sulfonic acid silica, which was also attributed to superior in‐pore mass transport and active site accessibility. The total acid number was reduced by 57 % over a 6 h reaction at 85 °C using the hierarchical PrSO_3_H/MM‐SBA‐15 catalyst. GC×GC–time‐of‐flight mass spectrometry (GC×GC–ToFMS) confirmed that catalytic upgrading removed almost all organic acids, and significantly lowered the concentration of reactive, phenolic, aldehyde, and ketone components, accompanied by the formation of carboxylic acids methyl esters and ethers.

## Experimental Section

Full details of the catalyst synthesis, bulk and surface characterization (TEM, XRD, N_2_ porosimetry, DRIFTS, XPS, TGA, pyridine adsorption/DRIFTS, propylamine adsorption/TGA‐MS), and catalytic esterification and bio‐oil analysis protocols are provided in the Supporting Information.

## Conflict of interest

*The authors declare no conflict of interest*.

## Supporting information

As a service to our authors and readers, this journal provides supporting information supplied by the authors. Such materials are peer reviewed and may be re‐organized for online delivery, but are not copy‐edited or typeset. Technical support issues arising from supporting information (other than missing files) should be addressed to the authors.

Supplementary
