# Supplementary material for: Impact of Macroporosity on Catalytic Upgrading of Fast Pyrolysis Bio‐Oil by Esterification over Silica Sulfonic Acids
Source: ChemSusChem. 2017 Aug 16;10(17):3506–11. doi: 10.1002/cssc.201700959 (PMC5638084; doi:10.1002/cssc.201700959)
Supplement: Supplementary file 1 — Supplementary [file CSSC-10-3506-s001.pdf]

CHEMISTRY & SUSTAINABILITY
CHEMSUSCHEM
ENERGY & MATERIALS

Supporting Information

Impact of Macroporosity on Catalytic Upgrading of Fast Pyrolysis Bio-Oil by Esterification over Silica Sulfonic Acids

Jinesh C. Manayil+,[a] Amin Osatiashtiani+,[a] Alvaro Mendoza,[a, b] Christopher M.A. Parlett,[a] Mark A. Isaacs,[a] Lee J. Durndell,[a] Chrysoula Michailof,[c] Eleni Heracleous,[c] Angelos Lappas,[c] Adam F. Lee,[a] and Karen Wilson*[a]

cssc_201700959_sm_miscellaneous_information.pdf

Author Contributions

J.M. Data curation: Lead; Formal analysis: Lead; Writing – original draft: Equal
A.O. Data curation: Lead; Formal analysis: Lead; Writing – original draft: Lead
A.M. Data curation: Supporting
C.P. Data curation: Supporting
L.D. Data curation: Supporting
M.I. Data curation: Supporting
C.M. Data curation: Supporting
E.H. Data curation: Supporting
A.L. Resources: Supporting
A.L. Conceptualization: Supporting; Funding acquisition: Supporting; Resources: Equal; Writing – review & editing: Lead
K.W. Conceptualization: Lead; Funding acquisition: Equal; Resources: Equal; Supervision: Lead; Writing – review & editing: Supporting.

Impact of macroporosity on catalytic upgrading of fast pyrolysis bio-oil by esterification over silica sulfonic acids

Jinesh C. Manayil [a], Amin Osatiashtiani [a], Alvaro Mendoza [a,b], Christopher M.A. Parlett [a], Mark A. Isaacs [a], Lee J. Durndell [a], Chrysoula Michailof [c], Eleni Heracleous [c], Angelos Lappas [c], Adam F. Lee [a], Karen Wilson [a]*

[a] European Bioenergy Research Institute, Aston University, Birmingham, B4 7ET, UK.
E-mail: k.wilson@aston.ac.uk

[b] Department of Chemical and Energy Technology, Universidad Rey Juan Carlos, C/Tulipán s/n, E-28933 Móstoles, Madrid, Spain.

[c] Chemical Process & Energy Resources Institute Centre for Research and Technology-Hellas, (CPERI/CERTH) 6th km Harilaou-Thermi Road, 57001,Thessaloniki, Greece.

Electronic Supporting Information

Experimental

Catalyst synthesis

Mesoporous and meso-macroporous catalysts were prepared adopting method reported elsewhere. SBA-15, with hexagonal cylindrical mesopores was synthesised via the original method of Zhao *et al.*[1] Typically, Pluronic P123 triblock copolymer (10 g) was dissolved in water (75 ml) and HCl solution (250 ml, 2M). The mixtures were stirred at 35 °C for dissolution and then tetraethyl orthosilicate (TEOS, 23 ml) was added with the synthesis maintained at 35 °C for 20 h under stirring. The resulting gel was then aged at 80 °C for 24 h. Finally, the solid product was filtered, washed with water and calcined under static air at 550 °C for 5 h with a ramp rate of 3 °C/min.

Meso-macroporous SBA-15 (MM-SBA-15) was synthesised via a modified SBA-15 synthesis which included a hard macropore template of polystyrene spheres. Polystyrene sphere were synthesised using the emulsion polymerisation method of Vandreuil and co-workers.[2] Potassium persulfate (0.16 g) was dissolved in distilled water (12 ml) at 70 °C. In a separate 500 ml three-necked round bottomed flask distilled water (377 ml) was purged under N_2 (10 ml min-1) at 70 °C. Styrene (50 ml) and divinylbenzene (9.5 ml) were each washed three times with sodium hydroxide solution (0.1 M, 1:1 vol/vol) followed by three washings with distilled water (1:1 vol/vol) to remove the polymerisation inhibitors. The washed organic phases were added to the purged water phase followed by the potassium persulfate solution. The mixture was left to stir under N_2 (10 ml min^{-1}) for 15 h, filtered and washed three times with distilled water (100 ml) and then three times with ethanol (100 ml). The final bead yield was in the region of 45 g. The SEM analysis (**Fig S1**) confirmed the formation of polystyrene with 250 nm bead size.

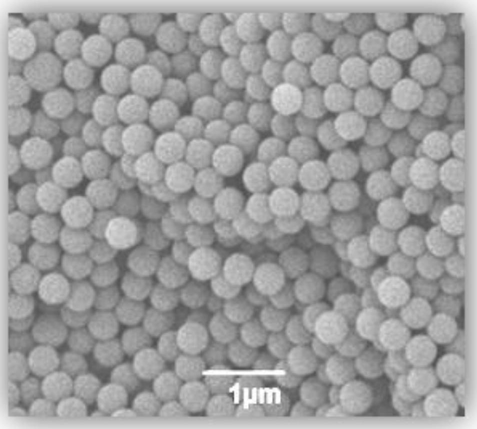

Fig S1. SEM micrographs of polystyrene beads synthesised.

The silica support was produced using the methodology published by Dhainaut and co-workers.[3] Pluronic P123 (10 g) was dissolved in water (75 ml) and hydrochloric acid (2 M, 290 ml) with stirring at 35 °C. Polystyrene beads (45 g) were added to the solution and left to stir for 1 h. Tetramethoxysilane (22.6 ml) was then added and left for 20 h with agitation. The resulting gel was aged under sealed conditions for 24 h at 80 °C under static conditions. The solid was filtered, washed with water (1000 ml) and dried at room temp before calcination at 550 °C for 6 h in air (ramp rate 0.5 °C min^{-1}). This yielded approximately 7.0 g of white solid.

Sulfonic acid functionalisation

Sulfonic acid functionalised silicas were prepared following our recent Hydrothermal Saline Promoted Grafting (HSPG) method.[4, 5] HSPG method gave high acid site loading and higher acidity than normal grafting method.[6, 7] Briefly, 1 g of each silica, namely SBA-15, or MM-SBA-15 and 200 mg NaCl were added to 30 ml H_2O and stirred for 15 min. Then, 1 ml of mercaptopropyl trimethoxysilane (MPTMS) was added to the solution. The mixture was then refluxed at 100 °C under stirring for 24 h, after which the resulting thiol-functionalized solid was filtered, washed three times with H_2O and dried at room temperature. Thiol groups were converted into $-SO_3H$ by mild oxidation using 30 ml of 30% hydrogen peroxide (Sigma-Aldrich) under stirring at room temperature for 24 h. The sulfonated solid product was subsequently filtered, washed three times with methanol and dried at room temperature. The resulting sulfonic acid-derivatised catalysts are denoted as $PrSO_3H$/SBA-15 and $PrSO_3H$/MM-SBA-15.

Catalyst characterisation

Physicochemical properties of catalysts were fully characterised. Surface areas and pore sizes were measured by N_2 physisorption on a Quantasorb Nova 4000 instruments, after sample outgassing at 120 °C for 4 h prior to analysis at -196 °C. Surface areas were calculated using the Brunauer–Emmett–Teller (BET) method over the range P/P_0 = 0.03–0.18, where a linear relationship was maintained. Pore size distributions were calculated using the Barrett–Joyner–Halenda (BJH) model applied to the desorption branch of the isotherm. Structural order was evaluated by means of low angle X-ray powder diffraction (XRD) on a Bruker D8 Advance diffractometer using the Cu K_α line in the range 2θ =0.6-5.0° with a step size of 0.02°, with phase identification evaluated by wide angle XRD in the range 2θ=10-80° with a step size of 0.04. Bulk sulphur loadings were calculated using CHNS using Thermo Scientific Flash 2000 CHNS-O analyser and verified by Thermogravimetric analysis (TGA). TGA was performed on a Mettler Toledo, TGA/DSC2 $Star^e$ system under N_2 flow during heating at 10 °C min^{-1} from 40–800 °C. Acid sites concentrations were measured by NH_3 pulse chemisorption using a Quantachrome ChemBET 3000 instrument interfaced to an MKS Minilab mass spectrometer (MS). Samples were degassed at 120 °C overnight under helium prior to NH_3 pulse titration at 100 °C. Temperature-programmed desorption (TPD) was subsequently performed on ammonia saturated samples between 100–500 °C.

Diffuse reflectance infra-red Fourier transform (DRIFT) spectra were obtained using a Thermo Scientific Nicolet environmental cell and smart collector accessory on a Thermo Scientific Nicolet iS50 FT-IR Spectrometer with MCT detector. Samples diluted homogeneously to 10 wt% with KBr were loaded in the environmental cell and subjected to evacuation at 200 °C for 2 h to remove physisorbed water/moisture. Analyses were performed at 200 °C. *Ex-situ* pyridine adsorption was performed by exposure of diluted samples (10 wt% in KBr) with neat pyridine. Excess physisorbed pyridine was removed in *vacuo* at 50 °C overnight prior to recording in *vacuo* Diffuse Reflectance Infra-red Fourier Transform (DRIFT) spectra at 50 °C in an environmental cell. Spectra were obtained using a Nicolet Avatar 370 MCT with Smart Collector accessory. Acid site loading was determined using Propylamine adsoption/TGA-MS measurement and further verified using NH_3 chemisorption. The catalysts were wetted with propylamine. Excess physisorbed propylamine was removed in vacuo at room temperature prior to temperature programmed desorption on a Mettler Toledo TGA/DSC 2 STAR^e System equipped with a Pfeiffer Vacuum ThermoStarTM GSD 301 T3 mass spectrometer. Acid sites concentrations were further measured by NH_3 pulse chemisorption using a Quantachrome ChemBET 3000 instrument interfaced to an MKS Minilab mass spectrometer (MS). Samples were degassed at 120 °C overnight under helium prior to NH_3 pulse titration.

Esterification of model molecules

Batch esterification of model compounds was performed using a Radleys Carousel Reactor Station at atmospheric pressure. In an optimised reaction condition, 150 mmol methanol, 5 mmol of linear carboxylic acids and 0.5 mmol of dihexyl ether (as an internal standard) were added to a glass round bottom flask under stirring at 60 °C. Catalyst was subsequently introduced (25 mg), and aliquots of the reaction mixture periodically withdrawn and filtered and diluted with dichloromethane for analysis on a GC (Varian 450-GC equipped with a Phenomenex ZB-5HT Inferno 15 m × 0.32 mm × 0.10 µm). In this study propanoic (C_3), hexanoic (C_6), and lauric (C_{12}) were screened as model molecules to check the influence of different kind of mesoporosity. All catalytic profiles are an average of 3 injections per sample. Conversions reported are based upon change in the concentration of the organic acid, with initial rates calculated over the first hour of reaction, wherein the conversion profile was linear. Turnover frequencies (TOFs) were determined from the initial reaction rate which was normalised to the acid site loadings as determined from propylamine/TG-MS analysis.

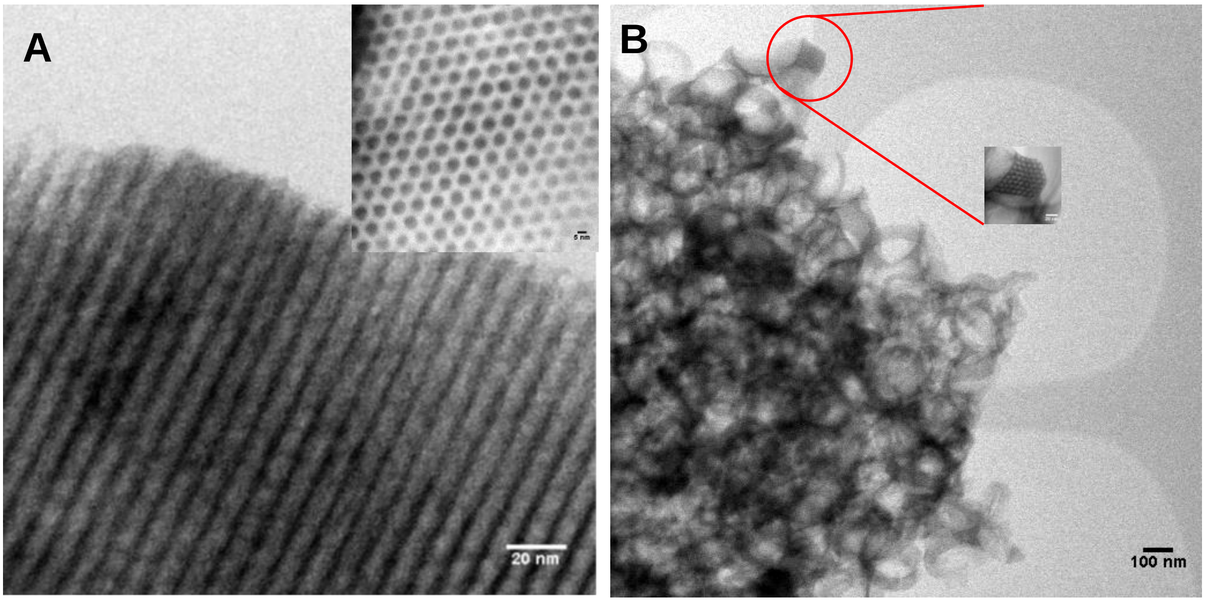

Fig S2. TEM of (A) mesoporous SBA-15 and (B) macro-meso SBA-15

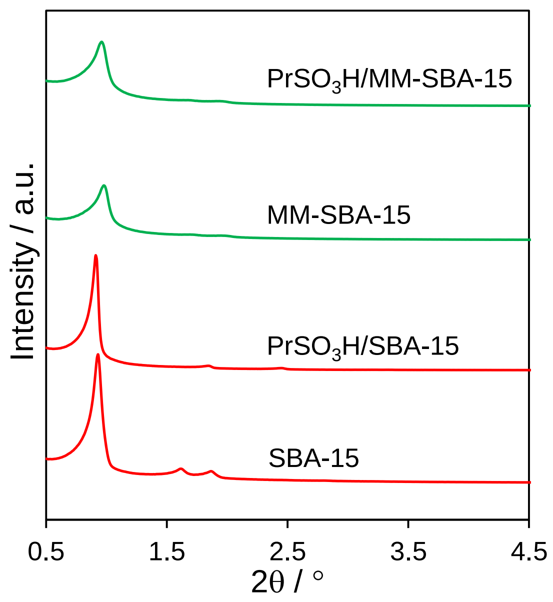

Fig S3. Low angle powder XRD pattern of SBA-15, $PrSO_3H$/SBA-15, MM-SBA-15, and $PrSO_3H$/MM-SBA-15.

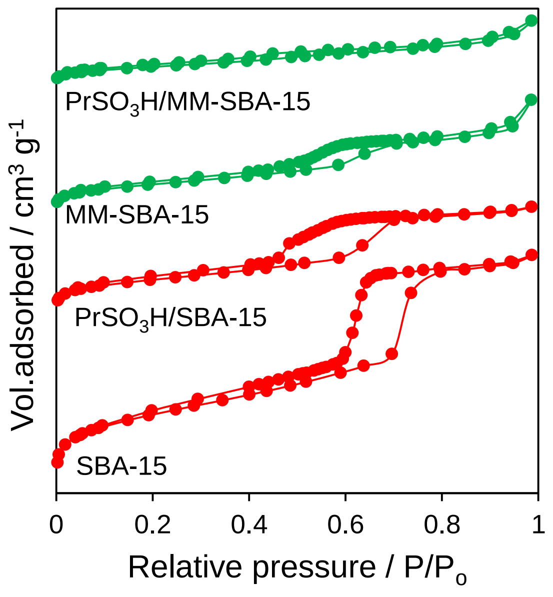

Fig S4. N_2 adsorption-desorption isotherm of SBA-15, $PrSO_3H$/SBA-15, MM-SBA-15, and $PrSO_3H$/MM-SBA-15.

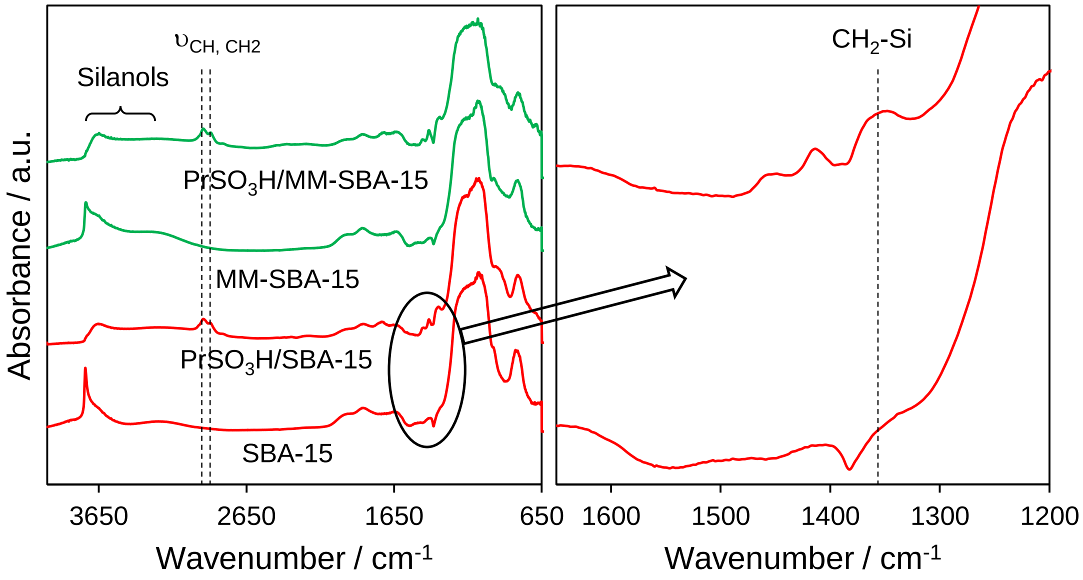

Fig S5. DRIFT spectra of SBA-15, $PrSO_3H$/SBA-15, MM-SBA-15, and $PrSO_3H$/MM-SBA-15.

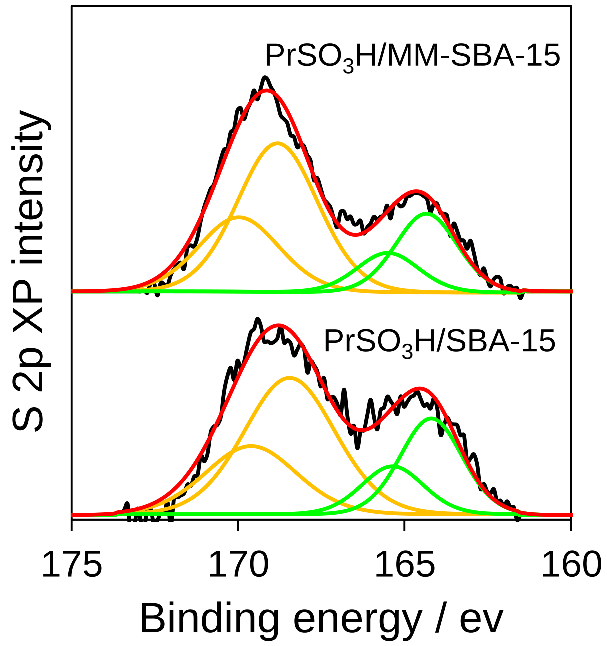

Fig S6. S 2p XP spectra of $PrSO_3H$/SBA-15 and $PrSO_3H$/MM-SBA-15, showing peaks for sulfur as thiol (164–165 eV) and in the oxidised sulfonic acid form (169–170 eV).

Table S1. Surface elemental analysis by XPS and percentage of thiol and sulfonic acid groups with respect to total sulfur content for functionalised meso and macro-mesoporous catalysts.

Catalyst	O / wt. %	C / wt. %	S / wt. %	Si / wt. %	$–SO_3H$ %	–SH %
$PrSO_3H$/SBA-15	37.5	18.6	2.0	42.0	83.4	16.6
$PrSO_3H$/MM-SBA-15	32.6	33.7	2.1	31.6	85.5	14.5

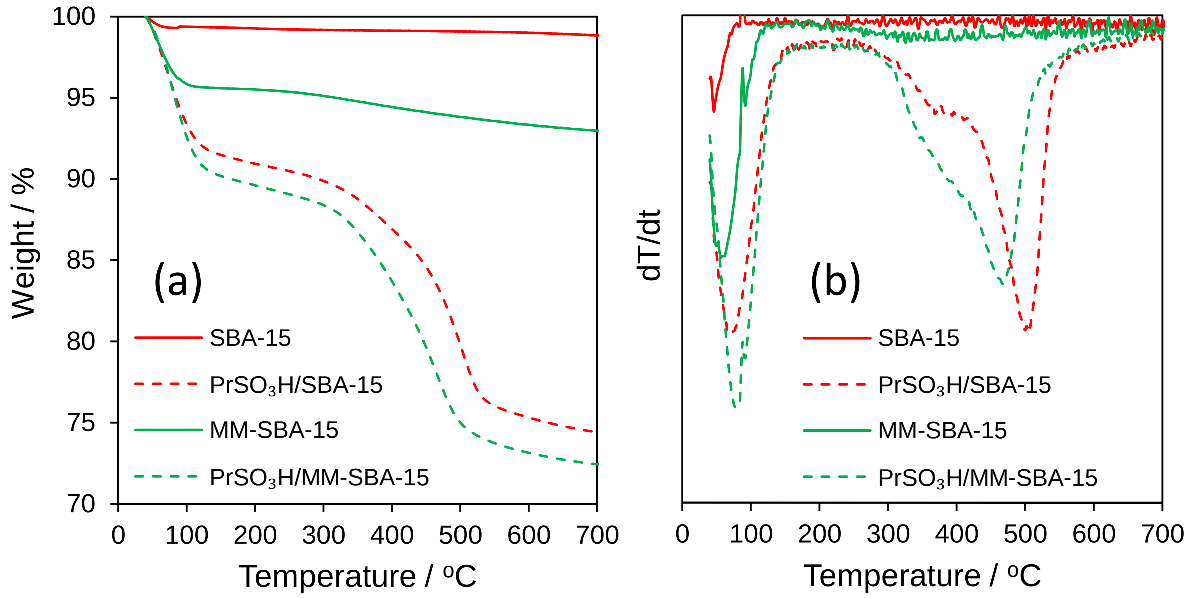

Fig S7. TGA (a) and differential thermogravimetric profiles (b) of SBA-15, $PrSO_3H$/SBA-15, MM-SBA-15, and $PrSO_3H$/MM-SBA-15, highlighting propyl sulfonic acid decomposition between 250-600 °C.

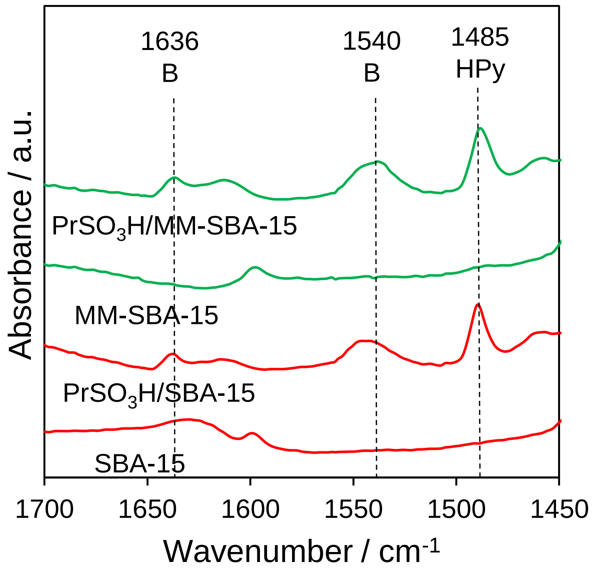

Fig S8. DRIFT spectra of pyridine adsorbed on SBA-15, $PrSO_3H$/SBA-15, MM-SBA-15, and $PrSO_3H$/MM-SBA-15.

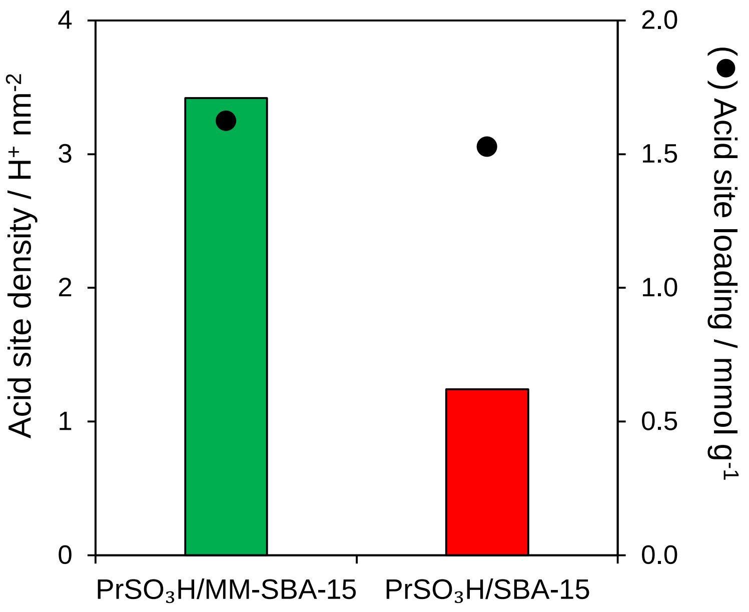

Fig S9. Acid site loading (dots) and acid site density (bars) for $PrSO_3H$/SBA-15 and $PrSO_3H$/MM-SBA-15.

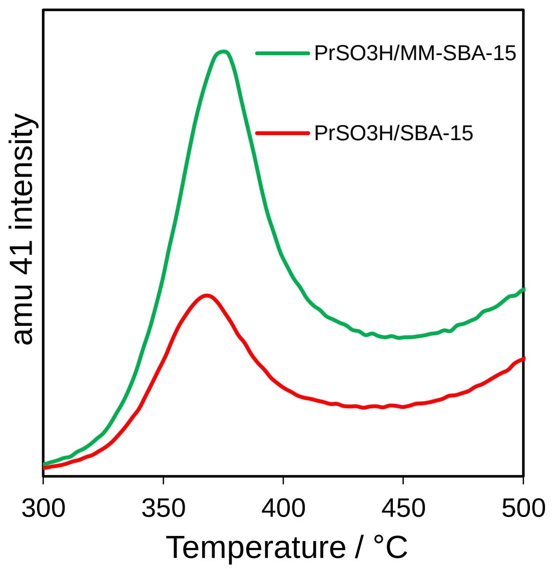

Fig S10. Propylamine TPD showing 41 amu mass spectrometer signal for reactively formed propene as a function of temperature for comparing the acid strength of $PrSO_3H$/SBA-15 and $PrSO_3H$/MM-SBA-15.

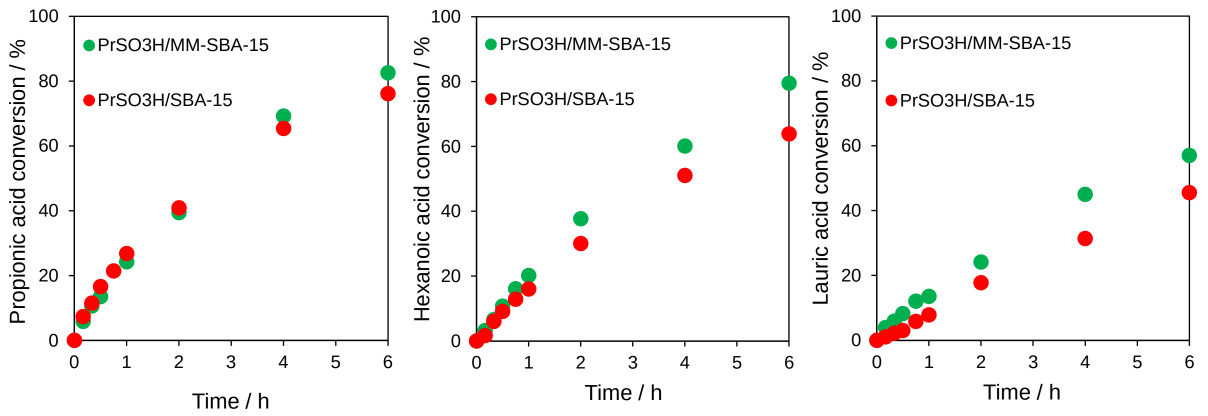

Fig S11. Esterification of carboxylic acids with methanol over $PrSO_3H/SBA-15$ and $PrSO_3H/MM-SBA-15$. (Reaction conditions: 25 mg catalyst, 5 mmol acid, acid:MeOH molar ratio= 1:30, 60 °C)

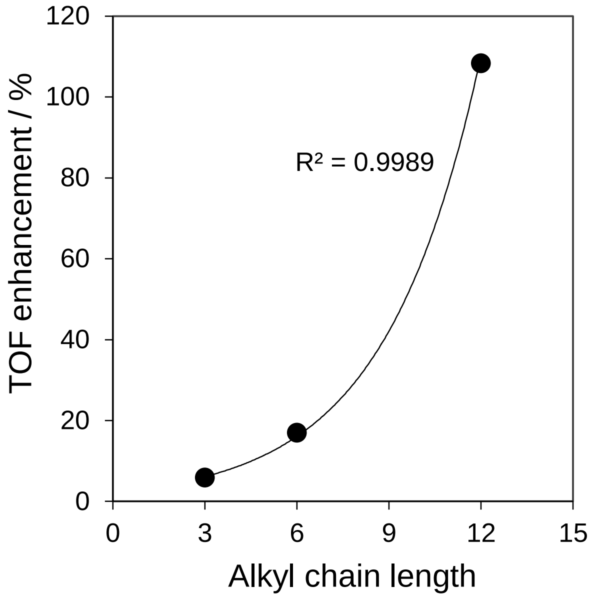

Fig S12. TOF enhancement over $PrSO_3H/MM-SBA-15$ with respect to $PrSO_3H/SBA-15$ as a function of carboxylic acid alkyl chain length.

Esterification of thermal pyrolysis bio-oil

Bio-oil from thermal pyrolysis of oak woodchips were provided by Centre for Research and Technology Hellas (CERTH). The main properties of the oak that was used as feedstock are shown in **Table S2**.

Table S2. Elemental analysis, moisture and ash content of the oak wood feedstock.

**Elemental analysis** (wt. %, dry biomass)				**Ash content** (wt. %, dry basis)	**Moisture content** (wt. %)	**Calorific value** (MJ kg^{-1})
**C**	**H**	**O**	**S**			
50.1	6.1	43.0	0.1	0.7	11.1	18.7

The reactor was loaded with silica sand and the bed temperature was constantly measured with a thermocouple. Once the reactor temperature reached 500 °C, the biomass was fed in the fluidized bed in a controlled manner with the use of a screw feeder at a feeding rate of 5 g min^{-1}. At the exit of the reactor, the bio-oil vapours passed through a filter fitted on the reactor exit to separate solid particles, and the vapours were then cooled to afford the liquid bio-oil. The gas products were vented through a wet test meter and analysed with a GC. The quantity of solid residue left in the reactor, deposited on the silica sand surface, consisted mainly of charcoal and coke-on-sand formed by thermal cracking, and was determined after in situ combustion at 650 °C and measurement of carbon in the produced gases (CO_2 and CO).

The bio-oil samples were submitted to elemental analysis and analysis of their density, viscosity, total acid number, water content and high heating value (HHV). The elemental analysis was conducted according to UOP 703 (ASTM D5291) method on a CHN-628 elemental analyser (LECO Corporation, USA). The bio-oil density was measured at 60 °C according to ASTM D 4052 and viscosity was measured at 50 °C according to ASTM D 445. The measurement of bio-oil acid content was carried out according to Modified D664A acid number titration method.[8] Briefly, 1 g of bio-oil was added into 100 ml ethylene glycol/water solution (95:5 vol:vol) and then the mixture was titrated with 0.1N KOH solution in 2-propanol. For the esterification of bio-oil, the equivalent mass of 10 mmol acid-containing bio-oil was mixed with 300 mmol methanol and the required amount of catalyst and the reaction was carried under reflux at 85 °C for 6 h and aliquots of the reaction mixture was withdrawn, filtered and titrated with similar method as explained before. The Karl-Fischer method, according to ASTM D 1744, was used for the determination of the aqueous content of the samples. For the High Heating Value (HHV), the method ASTM D 4809 was used. The results are presented in **Table S3**.

Table S3. Physicochemical characterisation of thermal fast pyrolysis bio-oil.

Property		Value
Calorific value / MJ kg^{-1}		18
H_2O content / wt. %		23
Total acid number / mg KOH g^{-1}		61.6
Elemental analysis / wt. %	C	43.7
	H	6.3
	O*	50.0
Viscosity at 50 °C / cSt		19
Density at 60 °C / g cm^{-3}		1.19

*by difference

Bio-oil analysis by GC×GC

For a more complete characterisation of their composition, the bio-oil samples were additionally analysed by GC×GC-ToFMS, after proper dilution in MeOH and without any other pretreatment. The GCGC analytical system was an Agilent 7890A GC with injector Agilent7683B series (Agilent Technologies, PaloAlto, CA, USA) connected to a Pegasus 4D time-of-flight mass spectrometer from Leco Instruments (St. Joseph, MI, USA). The first dimensional chromatographic separation was performed by an apolar column BPX-5 (5% phenyl polysilphenylene-siloxane) 30 m, I.D. 0.25 mm, df. 0.25 μm. The second dimensional column was situated in a secondary internal oven and was a BPX-50 (50% phenyl polysilphenylene-siloxane) 1.5 m, I.D. 0.1 mm, d.f. 0.1 μm, both from SGE Analytical Science Pty Ltd (Australia). Cryofocusing by liquid nitrogen and a quad jet dual stage modulator (Zoex, Houston, TX, USA) was applied. Instrument control, data acquisition and data processing were done by the ChromaToF (Leco) software. The ToFMS operated at an acquisition rate of 100 spectra/s and a mass range of m/z 45–400 amu. The modulation period was 5 s. The carrier gas (He grade 5) flow rate was 1 mL min^{-1}; split injection of 0.5 μL sample solution at a split ratio of 1:20 and an injection temperature of 250 °C. Temperature programming was performed at an initial temperature of 35 °C of the primary GC oven and was kept stable for 10 min. Then the temperature increased at a rate of 3 °C min^{-1} up to 250 °C, and afterwards with a rate of 15 °C min^{-1} up to the final temperature of 330 °C, where it was kept stable until the end of the program. Total run time was 102 min. The secondary oven was programmed 15 °C ahead of the primary GC oven gradient. Modulator temperature offset was 30 °C. The data acquisition and peak identification were based on the NIST05 library, using as minimum identification criteria similarity of 700 and S/N ratio of 50. The classification of the compounds was performed by borderline group type classification.[9]

Table S4. Detailed list of important compounds in the bio-oil before and after esterification.

**Phenolics peaks in the crude bio-oil**	**Area %**	**Phenolics peaks in esterified bio-oil using $PrSO_3H$/MM-SBA-15**	**Area %**
Phenol	0.97	Phenol	0.37
Phenol, 2-methyl-	0.40	Phenol, 2-methyl-	0.13
Phenol, 3-methyl-	0.67	Phenol, 3-methyl-	0.21
Phenol, 2-methoxy-	2.74	Phenol, 2-methoxy-	0.93
Phenol, 2,5-dimethyl-	0.63	Phenol, 2,5-dimethyl-	0.20
Phenol, 3-ethyl-	0.26	1,2-Benzenediol	1.91
2-Methoxy-6-methylphenol	0.07	Phenol, 2-methoxy-4-methyl-	0.79
1,2-Benzenediol	5.41	Phenol, 4-ethyl-2-methoxy-	0.09
Phenol, 2-methoxy-4-methyl-	2.42	1,2-Benzenediol, 4-methyl-	0.36
Phenol, 4-ethyl-3-methyl-	0.34	Eugenol	0.12
1,2-Benzenediol, 3-methyl-	0.27	Phenol, 2-methoxy-4-propyl-	0.10
Hydroquinone	0.46	4-Ethylcatechol	0.25
Phenol, 4-ethyl-2-methoxy-	0.82	Vanillin	0.60
1,2-Benzenediol, 4-methyl-	0.86	Phenol, 2-methoxy-4-(1-propenyl)-, (Z)-	0.08
2-Methoxy-4-vinylphenol	0.13	Ethanone, 1-(4-hydroxy-3-methoxyphenyl)-	0.36
2-Methyl-6-propylphenol	0.09		
Eugenol	0.42		
Phenol, 2-methoxy-4-propyl-	0.32		
4-Ethylcatechol	0.91		
Vanillin	1.80		
Phenol, 2-methoxy-4-(1-propenyl)-	0.18		
Phenol, 2-methoxy-4-(1-propenyl)-, (E)-	0.21		
Phenol, 2-methoxy-4-propyl-	0.48		
1,3-Benzenediol, 4-propyl-	0.38		
Ethanone, 1-(4-hydroxy-3-methoxyphenyl)-	1.13		
2-Propanone, 1-(4-hydroxy-3-methoxyphenyl)-	0.63		
Phenol, 4-(3-hydroxy-1-propenyl)-2-methoxy-	0.25		
4-Hydroxy-2-methoxycinnamaldehyde	0.34		
**Carbonyl peaks in the crude bio-oil**	**Area %**	**Carbonyl peaks in esterified bio-oil using $PrSO_3H$/MM-SBA-15**	**Area %**
Glycolaldehyde dimer	0.24	2(5H)-Furanone, 3-methyl-	0.34
2,3-Butanedione	0.27	1,2-Cyclopentanedione, 3-methyl-	0.36
Furfural	3.41	Benzaldehyde dimethyl acetal	0.07
2(5H)-Furanone	5.55	Furan, tetrahydro-2,5-dimethoxy	0.95
Benzaldehyde	0.11		
2(5H)-Furanone, 3-methyl-	0.85		
1,2-Cyclopentanedione, 3-methyl-	1.05		
2-Butanone, 3-methoxy-3-methyl-	0.23		
2,2-Dimethyl-3-heptanone	0.67		
2(5H)-Furanone, 4-methyl-	0.63		
2-Furancarboxaldehyde, 5-(hydroxymethyl)-	1.11		
Benzophenone	0.20		
Benzaldehyde, 4-(acetyloxy)-3-methoxy-	0.12		
Furan	0.09		
5-Hydroxymethyldihydrofuran-2-one	0.25		
**Ether peaks in the crude bio-oil**		**Ether peaks in esterified bio-oil using $PrSO_3H$/MM-SBA-15**	
Methyl-(2-hydoxy-3-ethoxy-benzyl)ether	0.17	Ethane, 1,1-dimethoxy-	0.22
		Ethane, 1,1,1-trimethoxy-	0.33
		Ethane, 1,1,2,2-tetramethoxy-	0.48
		Furan, 2-(2-ethoxy-1-methoxyethyl)-	0.10
		1,1,3-Trimethoxypropane	0.28
		Ethane, 1,1,2-trimethoxy-	0.14
		2-Propanol, 1,1-dimethoxy-	0.31

References

1. D. Zhao, J. Feng, Q. Huo, N. Melosh, G. H. Fredrickson, B. F. Chmelka and G. D. Stucky, *Science*, 1998, **279**, 548-552.
2. S. Vaudreuil, M. Bousmina, S. Kaliaguine and L. Bonneviot, *Advanced Materials*, 2001, **13**, 1310-1312.
3. J. Dhainaut, J.-P. Dacquin, A. F. Lee and K. Wilson, *Green Chemistry*, 2010, **12**, 296-303.
4. C. Pirez, A. F. Lee, J. C. Manayil, C. M. A. Parlett and K. Wilson, *Green Chemistry*, 2014, **16**, 4506-4509.
5. C. Pirez, M. T. Reche, A. F. Lee, J. C. Manayil, V. C. dos-Santos and K. Wilson, *Catal Lett*, 2015, **145**, 1483-1490.
6. J. C. Manayil, C. V. M. Inocencio, A. F. Lee and K. Wilson, *Green Chemistry*, 2016, **18**, 1387-1394.
7. J. C. Manayil, V. C. dos Santos, F. C. Jentoft, M. Granollers Mesa, A. F. Lee and K. Wilson, *ChemCatChem*, 2017, n/a-n/a.
8. S. H. Roby, M. Dutta, Y. Zhu and A. Pathiparampil, *Energy & Fuels*, 2015, **29**, 858-862.
9. C. Michailof, T. Sfetsas, S. Stefanidis, K. Kalogiannis, G. Theodoridis and A. Lappas, *Journal of Chromatography A*, 2014, **1369**, 147-160.
